# Rapidly progressive brain atrophy in septic ICU patients: a retrospective descriptive study using semiautomatic CT volumetry

**DOI:** 10.1186/s13054-021-03828-7

**Published:** 2021-11-29

**Authors:** Ryuta Nakae, Tetsuro Sekine, Takashi Tagami, Yasuo Murai, Eigo Kodani, Geoffrey Warnock, Hidetaka Sato, Akio Morita, Hiroyuki Yokota, Shoji Yokobori

**Affiliations:** 1 Department of Emergency and Critical Care Medicine, Nippon Medical School Hospital, 1-1-5, Sendagi, Bunkyo-ku, Tokyo, 113-8603 Japan; 2 Department of Radiology, Nippon Medical School Musashi Kosugi Hospital, 1-396, Kosugi-cho, Nakahara-ku, Kawasaki, Kanagawa 211-8533 Japan; 3 Department of Emergency and Critical Care Medicine, Nippon Medical School Musashi Kosugi Hospital, 1-396, Kosugi-cho, Nakahara-ku, Kawasaki, Kanagawa 211-8533 Japan; 4 Department of Neurosurgery, Nippon Medical School Hospital, 1-1-5, Sendagi, Bunkyo-ku, Tokyo, 113-8603 Japan; 5 PMOD Technologies Ltd., Sumatrastrasse 25, 8006 Zürich, Switzerland; 6 Graduate School of Medical and Health Science, Nippon Sport Science University, 1221-1, Kamoshida-cho, Aoba-ku, Yokohama, Kanagawa 227-0033 Japan

**Keywords:** Brain, Atrophy, Sepsis, Mechanical ventilation, Critical care outcomes

## Abstract

**Background:**

Sepsis is often associated with multiple organ failure; however, changes in brain volume with sepsis are not well understood. We assessed brain atrophy in the acute phase of sepsis using brain computed tomography (CT) scans, and their findings’ relationship to risk factors and outcomes.

**Methods:**

Patients with sepsis admitted to an intensive care unit (ICU) and who underwent at least two head CT scans during hospitalization were included (*n* = 48). The first brain CT scan was routinely performed on admission, and the second and further brain CT scans were obtained whenever prolonged disturbance of consciousness or abnormal neurological findings were observed. Brain volume was estimated using an automatic segmentation method and any changes in brain volume between the two scans were recorded. Patients with a brain volume change < 0% from the first CT scan to the second CT scan were defined as the “brain atrophy group (*n* = 42)”, and those with ≥ 0% were defined as the “no brain atrophy group (*n* = 6).” Use and duration of mechanical ventilation, length of ICU stay, length of hospital stay, and mortality were compared between the groups.

**Results:**

Analysis of all 42 cases in the brain atrophy group showed a significant decrease in brain volume (first CT scan: 1.041 ± 0.123 L vs. second CT scan: 1.002 ± 0.121 L, *t* (41) = 9.436, *p* < 0.001). The mean percentage change in brain volume between CT scans in the brain atrophy group was –3.7% over a median of 31 days, which is equivalent to a brain volume of 38.5 cm^3^. The proportion of cases on mechanical ventilation (95.2% vs. 66.7%; *p* = 0.02) and median time on mechanical ventilation (28 [IQR 15–57] days vs. 15 [IQR 0–25] days, *p* = 0.04) were significantly higher in the brain atrophy group than in the no brain atrophy group.

**Conclusions:**

Many ICU patients with severe sepsis who developed prolonged mental status changes and neurological sequelae showed signs of brain atrophy. Patients with rapidly progressive brain atrophy were more likely to have required mechanical ventilation.

**Supplementary Information:**

The online version contains supplementary material available at 10.1186/s13054-021-03828-7.

## Background

Advances in the treatment of sepsis have improved short-term outcomes and increased the survival rate [1, 2]. However, it has been reported that long-term outcomes have not improved [3–6]. A secondary analysis of the “A Controlled Comparison of Eritoran and placebo in patients with Severe Sepsis” (ACCESS) trial and the “Prospective Recombinant Human Activated Protein C Worldwide Evaluation in Severe Sepsis” (PROWESS)-SHOCK trial found that of those surviving after treatment for severe sepsis, 33.1% and 34.3% had not returned to independent living by 6 months, respectively [4–6]. Brinkman et al. [3] reported that most intensive care unit (ICU) patients were still at higher risk of death than the general population in the years after discharge, and that those patients admitted for acute renal failure or pneumonia had a mortality rate of > 40% in the 3 years after discharge. Recently, there have been several reports using magnetic resonance imaging (MRI) on the relationship between brain volume reduction and prognosis in patients receiving treatment in the ICU [7, 8]. Gunther et al. [7] found that in survivors treated in the ICU, the longer the duration of delirium, the smaller the brain volume up to 3 months after discharge, and smaller brain volume was associated with long-term cognitive impairment up to 12 months. Orhun et al. [8] reported that 16.1% of patients with sepsis-induced brain dysfunction had brain atrophy. Thus, it is important to evaluate changes in brain volume in the acute phase in critically ill patients; however, in clinical practice, MRI is difficult to obtain if the patient is on vasopressors or has unstable vital signs. In such a situation, images for neurological evaluation can only be obtained with head computed tomography (CT).

In this study, we examined the brain atrophy rate in the acute phase of sepsis using head CT scans, and its relationship to risk factors and outcomes.

## Methods

**Fig. 1:**
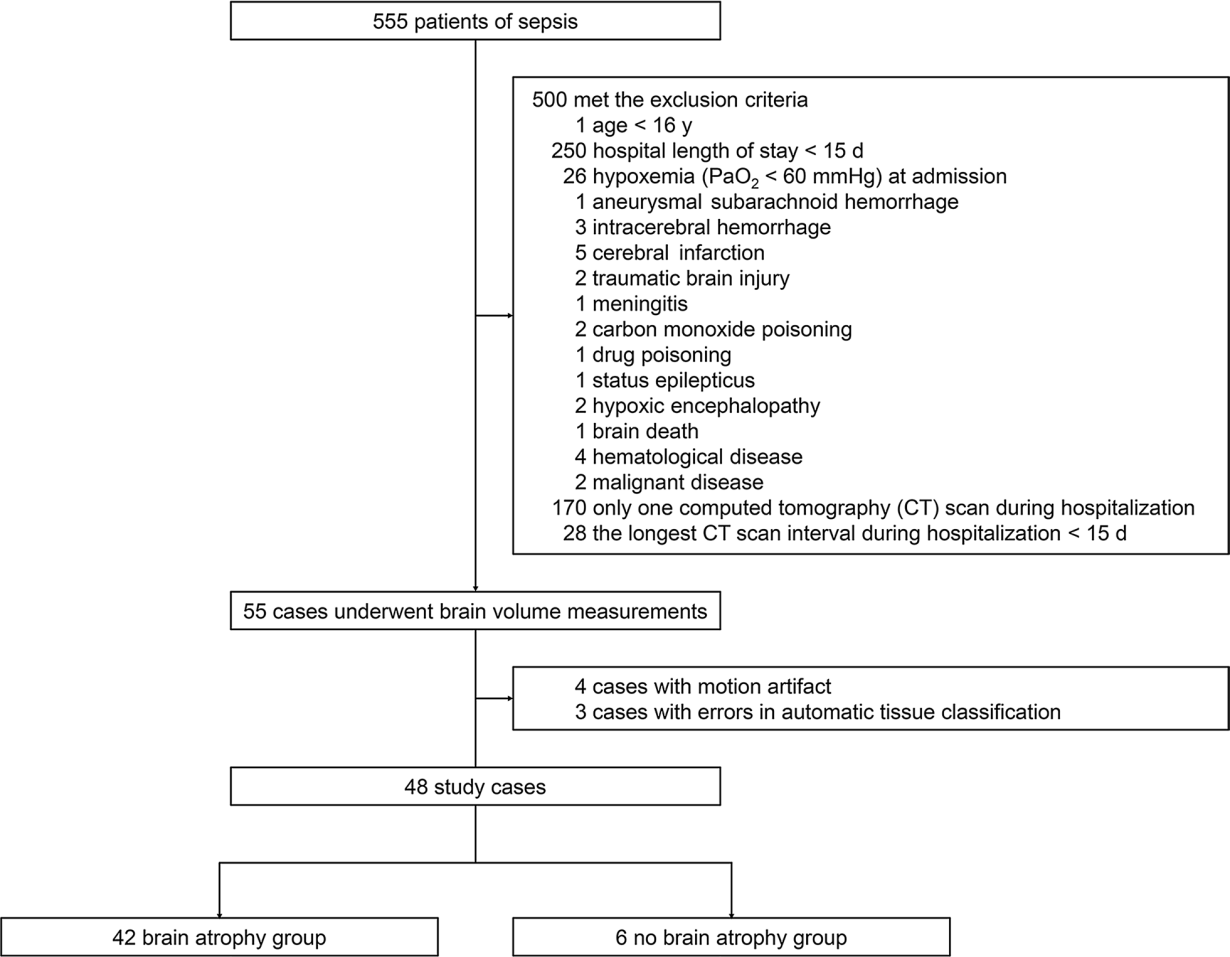
Flowchart of patients enrolled in the present study. Of 555 patients with sepsis managed from January 2015 to December 2020, 48 patients were enrolled

We retrospectively analyzed demographic, clinical, and radiologic data of 555 consecutive sepsis cases treated at the Department of Emergency and Critical Care Medicine, Nippon Medical School Hospital, from January 2015 to December 2020. Diagnosis of sepsis was based on the Japanese clinical practice guidelines for management of sepsis and septic shock 2016 (J-SSCG 2016) [9]. The J-SSCG2016 covers a total of 19 clinical areas and includes 89 clinical questions, and was developed to meet the needs of clinicians managing conditions including sepsis and disseminated intravascular coagulation (DIC). It was developed by 19 committee members and 52 working group members selected from the Japanese Society of Intensive Care Medicine and the Japanese Association for Acute Medicine (JAAM), in accordance with the procedures of the Medical Information Network Distribution Service (Minds). Exclusion criteria included age < 16 y; hospital length of stay < 15 d; comorbidities that may affect brain atrophy such as hypoxemia (PaO_2_ < 60 mmHg) at admission, aneurysmal subarachnoid hemorrhage, intracerebral hemorrhage, cerebral infarction, traumatic brain injury, meningitis, carbon monoxide poisoning, drug toxicity, status epilepticus, hypoxic encephalopathy, brain death, hematological disease, and malignant disease; only a single CT scan during hospitalization; and the longest CT scan interval during hospitalization < 15 d. This resulted in a total of 48 cases (Fig. 1).

We collected data on patient age; sex; past history of hypertension, diabetes mellitus, and atrial fibrillation; Glasgow Coma Scale (GCS) score, sequential organ failure assessment (SOFA) score [10], acute physiology and chronic health evaluation (APACHE) II score [11], and JAAM DIC score [12] at the time the diagnosis of sepsis was made; use of mechanical ventilation, vasopressor, heparin, antithrombin, and recombinant human soluble thrombomodulin (rhTM); and implementation of continuous renal replacement therapy (CRRT) and surgery.

### Management of sepsis

Treatment was administered immediately at the time of diagnosis based on J-SSCG 2016 [9]. In our institution, brain CT scans are routinely performed on septic patients at the time of admission (first CT scan). A second or further brain CT scans are obtained whenever prolonged disturbance of consciousness or abnormal neurological findings are observed. Here, when CT scans were acquired ≥ 3 times, the last CT scan acquired during hospitalization was adopted as the second CT scan. Treatment options such as heparin, antithrombin, rhTM, CRRT, and surgery were at the discretion of the treating physician.

### Evaluation of brain volume

**Fig. 2:**
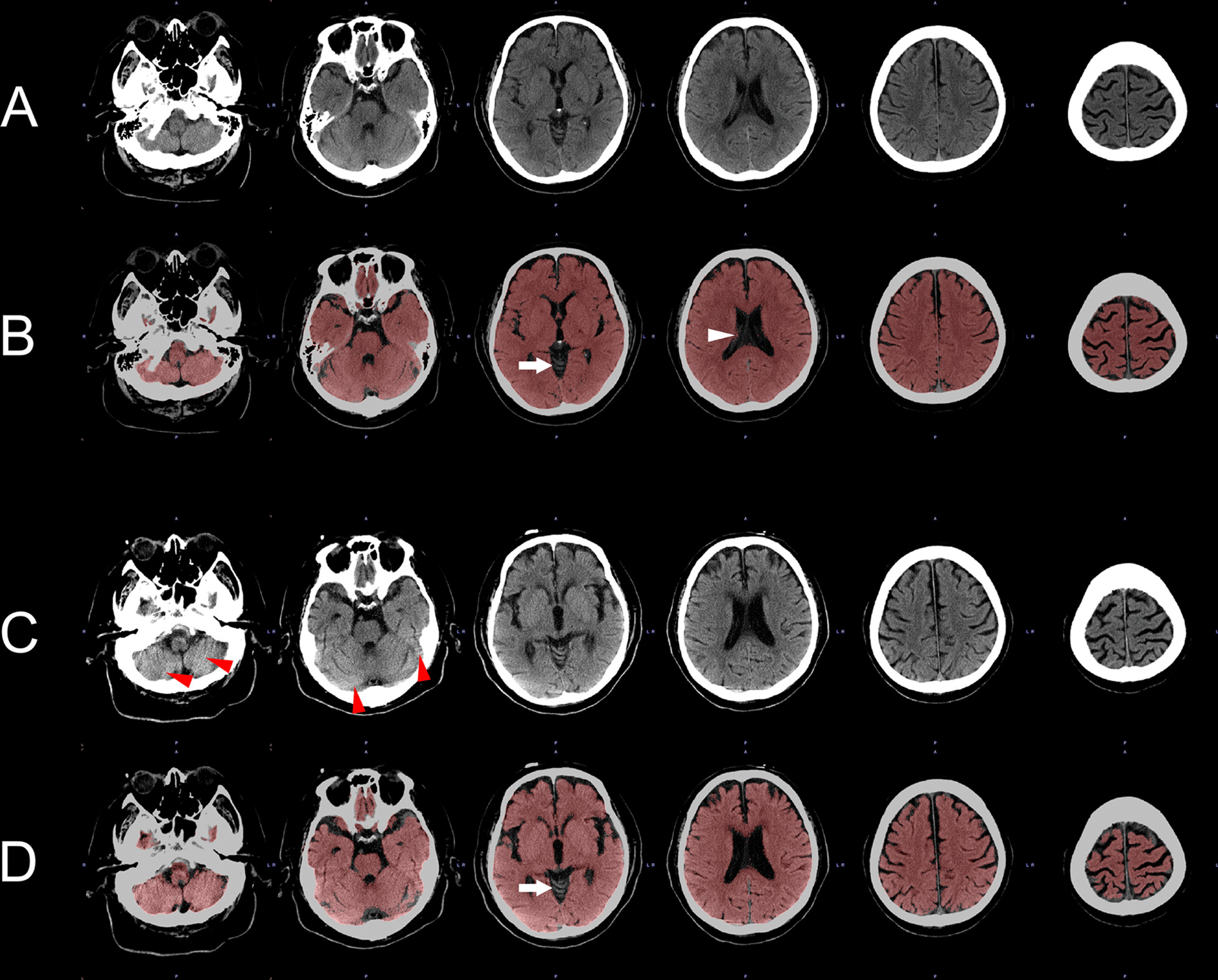
Axial view of brain computed tomography (CT) scan of a 63-year-old man who was hospitalized and treated for 107 days for sepsis due to soft tissue infection. **A** and **B** are the first CT scan and **C** and **D** are the second CT scan of the patient. **A** and **C** are original brain CT scans and **B** and **D** are brain CT scans with a corresponding segmentation map where gray and white matter are colored red. Compared to the first CT scan (**A**), atrophy is evident on the second CT scan (**C**). Although small misclassifications are observed in the cerebellar vermis (arrows on **B** and **D)** and the right choroid plexus (white arrowhead on **B)**, automatic segmentation was successful. Subtle beam-hardening artifact is found around the posterior fossa (red arrowheads on **C)**,; it did not affect the classification. A 7.4% decrease in brain volume was calculated by the automatic algorithm

Brain volume was automatically calculated from clinical routine CT images with 5 mm thick sections using commercial software (PMOD Version 4.002, PMOD Technologies Ltd., Zurich, Switzerland). We applied the methodology described by Adduru et al. [13, 14]. Briefly, intensity transformation was applied to original brain CT image data to enhance the tissue contrast between gray matter (GM), white matter (WM), and cerebrospinal fluid (CSF). Then, the scaled data were nonrigidly transformed to predefined template CT images as proposed by Rorden et al. [15]. As a result, the transformed images were registered to Montreal Neurological Institute (MNI) space. Six-compartment tissue segmentation using standard statistical parametric mapping (SPM) software Version 12 was adopted to separate GM, WM, CSF, bone, air, and other components according to the method of Ashburner and Friston [16]. The segmented images were then inversely transformed from MNI space to the original patients’ specific CT space. A volume of interest was drawn to include GM and WM to calculate the brain volume (Fig. 2). All steps were automatically performed without any user interaction.

After the calculation, one neuroradiologist (T.S.) validated the results of segmentation without any clinical information such as patient background or scan order (i.e. first or second). Seven datasets with segmentation error were excluded. Four had severe motion artifact either in the first or second CT scan and three had misclassification of CSF due to abnormal brain morphology.

### Group classification

After assessing for change in brain volume between the first and second CT scans, we assigned patients with brain volume change < 0% from the first CT scan to the second CT scan to the “brain atrophy group (*n* = 42)” and those with ≥ 0% to the “no brain atrophy group (*n* = 6).” The demographic characteristics of each group were compared, including the need for and length of time on mechanical ventilation, length of ICU stay, length of hospital stay, mortality, prevalence of delirium as assessed using the confusion assessment method for the intensive care unit (CAM-ICU) [17] until second CT scan, and Medical Research Council (MRC) score. The MRC score, ranging from 0 to 60, was assessed during early awakening in surviving patients as an indicator of ICU-acquired weakness (ICU-AW) using six prespecified muscle groups [18]. CAM-ICU assessments were performed every 12 h by nurses, and MRC scores were assessed by physical therapists for muscle strength and scored by physicians.

### Statistical analysis

Data are expressed as number (%), mean (standard deviation: SD), or median (interquartile range: IQR). Continuous variables were compared between groups using Student’s *t*-test or the Mann–Whitney *U*-test, and categorical variables using the *χ*^2^ test. A value of *p* < 0.05 was considered statistically significant. All statistical analyses were performed using commercial software (SPSS Version 25.0®; IBM Corp., Armonk NY, USA).

## Results

**Fig. 3:**
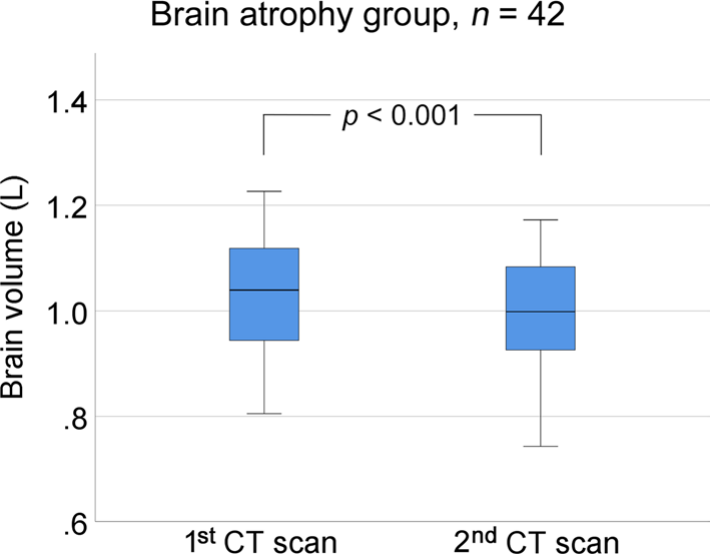
Change in brain volume in the 42 cases in the brain atrophy group from the first computed tomography (CT) scan to the second CT scan

Analysis of all 42 cases in the brain atrophy group showed a significant decrease in brain volume (first CT scan: 1.041 ± 0.123 L vs. second CT scan: 1.002 ± 0.121 L, *t* (41) = 9.436, *p* < 0.001) (Fig. 3). Brain atrophy was diffuse, and not focal, in all cases.

Demographic and clinical characteristics of patients are summarized in Table 1. Median (IQR) of SOFA score and APACHE II score of the eligible cases were as high as 13 (11–15) and 27 (21–31), respectively, indicating that many cases with severe sepsis were included in the study. In addition, 39 (81.3%) cases met the DIC diagnostic criteria of JAAM DIC score ≥ 4. The groups were comparable with regard to age; sex; presence of medical histories such as hypertension, diabetes mellitus, and atrial fibrillation; and clinical scores such as GCS score, SOFA score, APACHE II score, and JAAM DIC score. The incidence of mechanical ventilation use was significantly higher in the brain atrophy group than in the no brain atrophy group (95.2% vs. 66.7%, *p* = 0.02) (Table 1).

**Table 1:** Characteristics of the study population

Characteristic	Total (*n* = 48)	Brain atrophy group (*n* = 42)	No brain atrophy group (*n* = 6)	*p*
*Demographics*				
Age, median (IQR), years	73 (63–80)	73 (63–79)	79 (68–84)	0.26
Male, *n* (%)	31 (64.6)	28 (66.7)	3 (50.0)	0.43
*Medical history*				
Hypertension, *n* (%)	16 (33.3)	13 (31.0)	3 (50.0)	0.36
Diabetes mellitus, *n* (%)	14 (29.2)	13 (31.0)	1 (16.7)	0.48
Atrial fibrillation, *n* (%)	10 (20.8)	9 (21.4)	1 (16.7)	0.79
*Clinical score*				
GCS score, median (IQR)	12 (8–14)	13 (9–14)	10 (3–13)	0.18
SOFA score, median (IQR)	13 (11–15)	14 (11–15)	11 (9–15)	0.29
APACHE II score, median (IQR)	27 (21–31)	27 (24–31)	28 (16–31)	0.63
JAAM DIC score, median (IQR)	6 (4–7)	6 (4–7)	5 (3–6)	0.26
*Treatment*				
Mechanical ventilation, *n* (%)	44 (91.7)	40 (95.2)	4 (66.7)	**0.02**
Vasopressors, *n* (%)	38 (79.2)	35 (83.3)	3 (50.0)	0.06
Heparin, *n* (%)	5 (10.4)	5 (11.9)	0 (0.0)	0.38
Antithrombin, *n* (%)	15 (31.3)	13 (31.0)	2 (33.3)	0.91
rhTM, *n* (%)	13 (27.1)	12 (28.6)	1 (16.7)	0.54
CRRT, *n* (%)	37 (77.1)	34 (81.0)	3 (50.0)	0.10
Surgery, *n* (%)	12 (25.0)	12 (28.6)	0 (0.0)	0.14

Significant value is given in bold

Table 2 shows the brain volumes and other outcomes. The mean percentage change of brain volume between CT scans in the brain atrophy group was –3.7% (equivalent to a brain volume of 38.5 cm^3^) over a median of 31 days. The mortality rate did not significantly differ between the two groups. In patients on mechanical ventilation, the median length of mechanical ventilation was significantly higher in the brain atrophy group than in the no brain atrophy group (28 [IQR: 15–57] days vs. 15 [IQR: 0–25] days, *p* = 0.04) (Table 3). Excluding the 12 patients who remained comatose until the second CT scan or with a history of severe dementia, the incidence of delirium in both groups reached 66.7% (Table 4). Among patients without limb paralysis, the median MRC scores decreased substantially from the full score of 60 in both groups, suggesting muscle weakness, but with no significant difference between the two groups (Table 5).

**Table 2:** Clinical results

	Total (*n* = 48)	Brain atrophy group (*n* = 42)	No brain atrophy group (*n* = 6)	*p*
*Radiological findings*				
Brain volume at 1st CT scan, mean ± SD, L	1.033 ± 0.120	1.041 ± 0.123	0.975 ± 0.072	0.16
Brain volume at 2nd CT scan, mean ± SD, L	1.000 ± 0.114	1.002 ± 0.121	0.986 ± 0.064	0.80
Percent change of brain volume, mean ± SD, %	–3.1 ± 2.9	–3.7 ± 2.6	1.2 ± 1.4	** < 0.001**
Number of days between CT scans, median (IQR), days	29 (18–46)	31 (19–49)	20 (17–23)	0.07
*Length of stay/mortality*				
Length of ICU stay, median (IQR), days	28 (15–45)	28 (15–48)	17 (3–31)	0.15
Length of hospital stay, median (IQR), days	41 (28–70)	45 (28–72)	30 (25–54)	0.29
Mortality, *n* (%)	14 (29.2)	11 (26.2)	3 (50.0)	0.24

Significant value is given in bold

**Table 3:** Time on mechanical ventilation and brain atrophy rate

	Total (*n* = 44)	Ventilated brain atrophy group (*n* = 40)	Ventilated no brain atrophy group (*n* = 4)	*p*
Length of time on mechanical ventilation, median (IQR), days	27 (14–52)	28 (15–57)	15 (0–25)	**0.04**

Significant value is given in bold

**Table 4:** Prevalence of delirium until second CT scan

	Total (*n* = 36)	Brain atrophy group (*n* = 33)	No brain atrophy group (*n* = 3)	*p*
Delirium, *n* (%)	24 (66.7)	22 (66.7)	2 (66.7)	1.00

**Table 5:** Medical Research Council score in surviving patients

	Total (*n* = 31)	Brain atrophy group survivors (*n* = 28)	No brain atrophy group survivors (*n* = 3)	*p*
MRC score, median (IQR)	36 (24–48)	33 (24–48)	48 (36–60)	0.09

## Discussion

We found significant brain atrophy in patients with severe sepsis with a mean decrease in brain volume of − 3.7% (38.5 cm^3^) over a median of 31 days. Further brain atrophy was associated with the use and length of time on mechanical ventilation.

We have often observed the occurrence of brain atrophy in septic patients during a relatively short period of hospitalization (Additional file 1, 2: Figs. S1 and S2). Cerebral infarction due to hypoperfusion or DIC in septic patients has been reported [19, 20]. Although impaired cerebral blood flow (CBF) may be responsible for the atrophy observed in the present study, the mechanism is considered to differ from that with common cerebral infarction because there were no cases with new low-density areas on CT scans, and the atrophy was observed diffusely rather than focally. Considering that 35 (83.3%) of the 42 patients in the brain atrophy group were diagnosed with DIC with a JAAM DIC score ≥ 4 and that these patients were in a hypercoagulable state [21], diffuse atrophic change may have progressed due to diffuse infarction caused by microthrombi that were not reflected on CT scan. Chang [22] stated that in patients with sepsis, endotheliopathy is caused by the membrane-attack complex generated by complement activation, resulting in microthrombus formation and multiorgan dysfunction syndrome (MODS) caused by disseminated intravascular microthrombosis. We speculate that the same mechanism may be responsible for the occurrence of diffuse cerebral atrophy observed in this study as one of the MODS.

Our findings also suggest that rapidly progressive cerebral atrophy in septic patients was associated with mechanical ventilation. Although mechanical ventilation has the effect of improving oxygenation and alveolar recruitment, the increase in intrathoracic pressure caused by positive end-expiratory pressure (PEEP) can increase jugular venous pressure and inhibit cerebral venous outflow [23]. Impaired venous outflow leads to increased cerebral blood volume (CBV), resulting in increased intracranial pressure (ICP), potentially decreasing CBF [24, 25]. In addition, in patients with hemodynamic instability, PEEP up to 20 cm H_2_O has been shown to significantly decrease systemic venous return, leading to decreased cardiac output, which in turn decreases systemic blood pressure and cerebral perfusion pressure (CPP), decreasing CBF [26]. This mechanism is known as the Starling resistance model and represents the role of damaged venous outflow in the ICP response to PEEP [27]. This may act in concert with the cerebral hypoperfusion caused by sepsis. Recent animal studies have also suggested that brain atrophy associated with short-term mechanical ventilation may be caused by reduced amyloid-β clearance associated with brain outflow impairment [28].

Previous reports have shown that decreased brain volume is causally related to decreased motor and cognitive functions [29–32]. Kido et al. [30] reported that atrophy in the left cerebellum and left prefrontal cortex was associated with reduced walking speed, while atrophy in the right putamen, right posterior superior parietal cortex, and cerebellum were associated with balance difficulties. Gado et al. [29] reported that volume expansion of the CSF space on CT was a highly sensitive indicator for detecting cognitive ability. Therefore, it is possible that brain atrophy is a factor in the development of cognitive and physical impairment. Adduru et al. [13] reported that the Alzheimer’s disease group had 1.7% lower brain volume than the control group using an automatic segmentation method with CT scans. An MRI study by Tabatabaei-Jafari et al. [33] showed that the rate of overall brain atrophy in patients with newly diagnosed Alzheimer’s disease ranged from 1.83 to 2.71% per year. These results suggest that the 3.7% reduction in brain volume in this study is not negligible. Inflammation, oxidative damage, and neuronal apoptosis associated with sepsis have also been reported to cause neuronal damage and brain dysfunction [34, 35] and these changes have been observed in patients with long-term cognitive impairment [36].

Gunther et al. [7] found that in ICU-treated survivors, longer duration of delirium was associated with smaller brain volume up to 3 months post-discharge. As a mechanism, some studies have reported that regional cerebral blood flow in the frontal, temporal, and occipital cortices, and in the thalamus, basal ganglia, and pons is greatly reduced during acute delirium [37, 38]. In our study, the number of patients with delirium was also high, at 66.7% of all patients, but there was no significant difference between the brain atrophy and the no brain atrophy groups. In addition, Harvey et al. [39] showed that ICU-AW developed in 25%–80% of people who are ventilated for > 4 d and in 50–75% of people with sepsis. Similar to their study, the MRC score as an assessment of motor function was substantially decreased in the target patients in this study, but there was no significant difference between the brain atrophy group and the no brain atrophy group. One reason that these investigations did not show differences may be that the size of the no brain atrophy group was so small.

Although several studies have evaluated brain volume changes in septic patients using MRI [7, 8], no study has evaluated brain volume changes using CT scans. We performed automatic brain segmentation to calculate brain volume from clinical routine CT images with 5 mm thick sections. The standard method of brain volumetry uses high-resolution MRI as input [40]. However, in the clinical setting, MRI is difficult to acquire when patients are on vasopressors and have unstable vital signs. In that situation, we can obtain only brain CT images for neurological assessment. In segmentation of the brain, the low tissue contrast in CT hampers the objective evaluation of brain volume. To overcome this drawback, CT-based brain-volume calculation utilizing pre-defined CT-template images has been developed [15]. Compared to the gold-standard MRI-based volume estimates, this approach is sensitive enough to yield brain volume estimates within 3–5% [14]. The clinical utility of CT brain volumetry has already been validated in the discrimination of Alzheimer’s disease from controls [13]. We excluded three patients due to errors in automatic tissue classification. This rate (5.8%, 3/51) is not inferior to the previous reports (11.2%, 17/152) [13].

The strength of this study is that we used automatic CT brain volumetry to quantitatively assess brain volume. This technique requires only a computational cost of about 15 min per patient dataset and does not require an experienced operator. As CT data are expressed in the same standardized values (Hounsfield units) among different scanners, this method can be accurately applied to any CT scanner in multiple institutions. This automatic approach would likely be highly useful in the conduct of a much larger/multi-center cohort.

There are several limitations to our study. First, it was conducted as a retrospective study and selection bias was inevitable. Of the 555 patients with sepsis, only 48 cases met the inclusion criteria. The prolonged disturbance of consciousness and abnormal neurological findings that triggered the CT scan may be related to brain atrophy, and may have overdetected patients who presented with brain atrophy. However, the short time between scans would eliminate any contribution of age-related atrophy. Further large prospective studies are needed to validate our results. Second, since this study included patients who had had multiple head CT scans, most of the patients presented with severe sepsis or DIC complications. We found that patients with severe sepsis had a higher incidence of brain atrophy, but this is not considered to be generalizable to sepsis. Third, since this study was conducted on septic patients, we could not compare brain volumes with those of non-septic patients. Therefore, the results of this study may not indicate that rapidly progressive brain atrophy is a change specific to sepsis. Fourth, we did not investigate cognitive decline associated with brain atrophy. Finally, multivariate analysis was not performed because it could be assumed that the statistical model would be unstable.

## Conclusions

Many ICU patients with severe sepsis who developed prolonged mental status changes and neurological sequelae showed signs of brain atrophy. Patients with rapidly progressive brain atrophy were more likely to have required mechanical ventilation.

## Supplementary Information

**Additional file 1**: **Fig. S1.** Axial brain computed tomography (CT) sections of a 60-year-old man, who was hospitalized and treated for 60 days for sepsis due to lung abscess. **A** Brain CT scan on admission. **B** Brain CT scan obtained 38 days after admission, revealing enlarged lateral ventricles (arrows) and bilateral frontal and temporal lobe cortical sulci (arrowheads).

**Additional file 2**: **Fig. S2.** Axial brain computed tomography (CT) sections of a 65-year-old man, who was hospitalized and treated for 109 days for sepsis due to bacterial pneumonia. **A** Brain CT scan on admission. **B** Brain CT scan obtained 65 days after admission, revealing enlarged bilateral frontal and temporal cortical sulci without change in the size of the lateral ventricles (arrowheads).

## Data Availability

The datasets supporting the conclusions of this article will be provided based on reasonable request to the corresponding author.

## Abbreviations

APACHE: Acute physiology and chronic health evaluation
CAM-ICU: Confusion assessment method for the intensive care unit
CBF: Cerebral blood flow
CBV: Cerebral blood volume
CPP: Cerebral perfusion pressure
CRRT: Continuous renal replacement therapy
CSF: Cerebrospinal fluid
CT: Computed tomography
DIC: Disseminated intravascular coagulation
GCS: Glasgow Coma Scale
GM: Gray matter
ICP: Intracranial pressure
ICU-AW: Intensive care unit-acquired weakness
ICU: Intensive care unit
IQR: Interquartile range
J-SSCG: Japanese clinical practice guidelines for management of sepsis and septic shock
JAAM: Japanese association for acute medicine
MNI: Montreal Neurological Institute
MODS: Multiorgan dysfunction syndrome
MRC: Medical Research Council
MRI: Magnetic resonance imaging
PEEP: Positive end-expiratory pressure
rhTM: Recombinant human soluble thrombomodulin
SD: Standard deviation
SOFA: Sequential organ failure assessment
SPM: Statistical parametric mapping
WM: White matter
