# Supplementary figures and images for: Rapidly progressive brain atrophy in septic ICU patients: a retrospective descriptive study using semiautomatic CT volumetry
Source: Crit Care. 2021 Nov 29;25:411. doi: 10.1186/s13054-021-03828-7 (PMC8628398; doi:10.1186/s13054-021-03828-7)

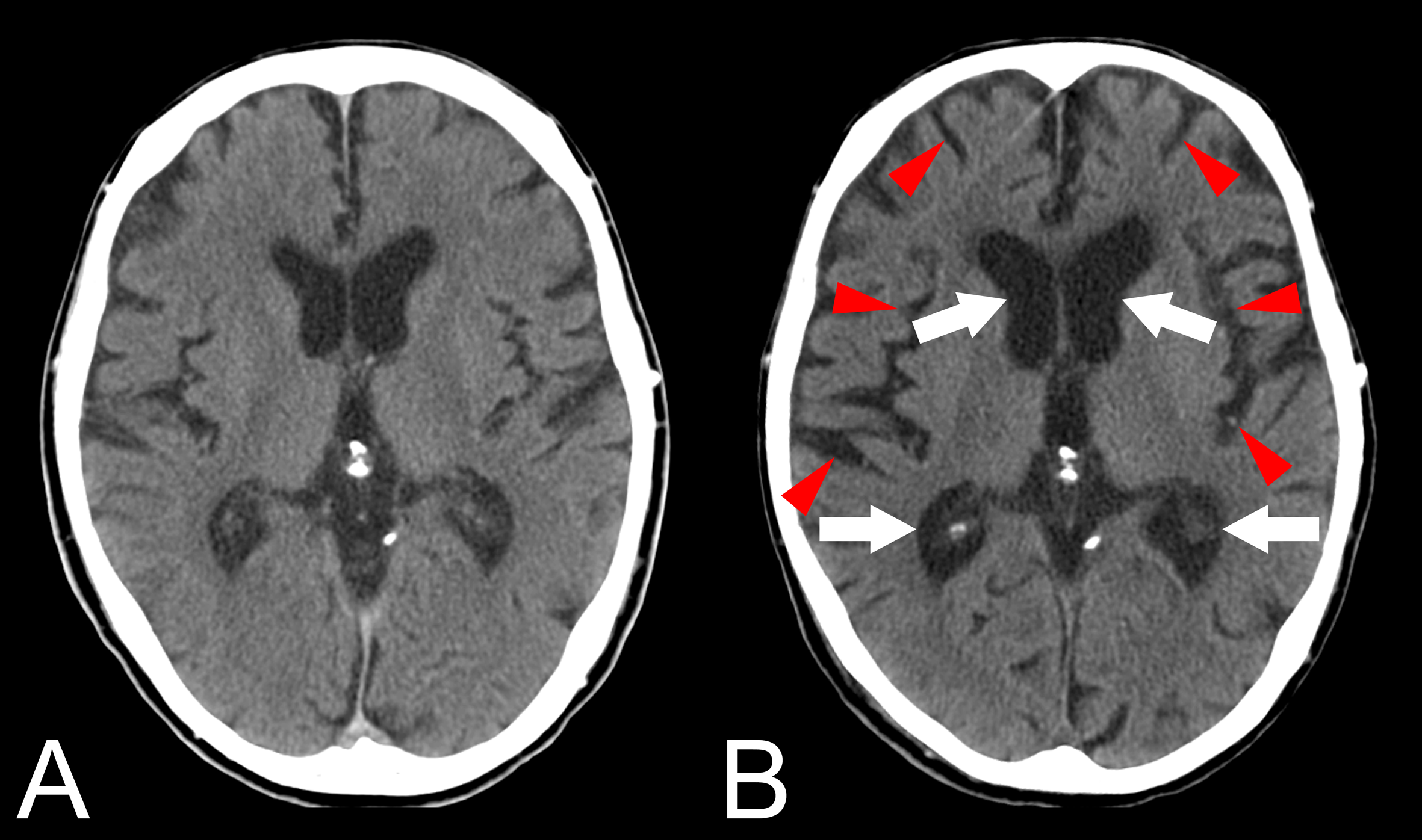

Supplement: Supplementary file 1 — Additional file 1: Fig. S1. Axial brain computed tomography (CT) sections of a 60-year-old man, who was hospitalized and treated for 60 days for sepsis due to lung abscess. A Brain CT scan on admission. B Brain CT scan obtained 38 days after admission, revealing enlarged lateral ventricles (arrows) and bilateral frontal and temporal lobe cortical sulci (arrowheads). [file 13054_2021_3828_MOESM1_ESM.tif]

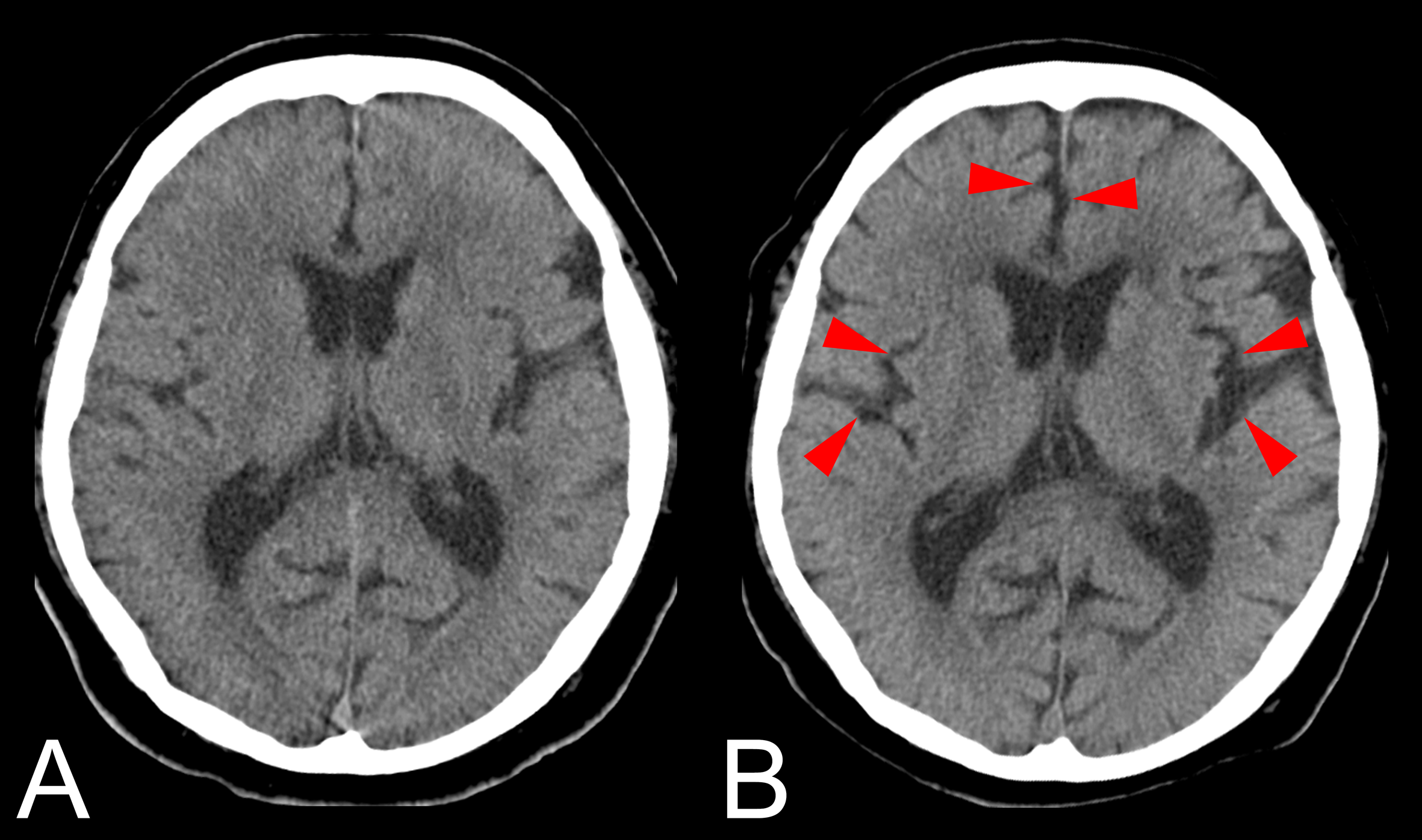

Supplement: Supplementary file 2 — Additional file 2: Fig. S2. Axial brain computed tomography (CT) sections of a 65-year-old man, who was hospitalized and treated for 109 days for sepsis due to bacterial pneumonia. A Brain CT scan on admission. B Brain CT scan obtained 65 days after admission, revealing enlarged bilateral frontal and temporal cortical sulci without change in the size of the lateral ventricles (arrowheads). [file 13054_2021_3828_MOESM2_ESM.tif]
